# Cost-Utility Analysis of Endovascular Ultrasound Renal Denervation to Treat Resistant Hypertension in the United States

**DOI:** 10.1016/j.jscai.2025.103601

**Published:** 2025-06-17

**Authors:** Rod S. Taylor, Kieran Murphy, Noam Kirson, Jack Pfefferkorn, Ajay J. Kirtane, Michel Azizi, Peter Neumann

**Affiliations:** a MRC/CSO Social and Public Health Sciences Unit, University of Glasgow, Glasgow, United Kingdom; b Robertson Centre for Biostatistics, University of Glasgow, Glasgow, United Kingdom; c ReCor Medical, Palo Alto, California; d Analysis Group Inc, Boston, Massachusetts; e NewYork-Presbyterian Hospital/Columbia University Irving Medical Center, New York, New York; f Cardiovascular Research Foundation, New York, New York; g Université Paris Cité, INSERM CIC1418, Paris, France; h AP-HP, Hôpital Européen Georges-Pompidou, Hypertension Department, and DMU CARTE, Paris, France; i Center for the Evaluation of Value and Risk in Health, Tufts Medical Center, Boston, Massachusetts

**Keywords:** cardiovascular disease, cost-effectiveness, hypertension, renal denervation, resistant hypertension, ultrasound

## Abstract

**Background:**

This study evaluated the cost utility of ultrasound renal denervation (uRDN) for resistant hypertension in the United States.

**Methods:**

A previously published Markov model was adapted to compare total costs and quality-adjusted life years (QALY) between uRDN plus standard of care (SoC) vs SoC alone over a lifetime horizon from a US health care system perspective. Patient characteristics and clinical inputs were drawn from the RADIANCE-HTN TRIO trial, and the incidence of key cardiovascular events was estimated using published risk equations. Utility values and US health care cost inputs were based on a targeted literature review. Incremental cost-effectiveness ratio was evaluated against standard cost-effectiveness thresholds ranging from $50,000 to $100,000 per QALY. Scenario, deterministic, and probabilistic sensitivity analyses were used to assess the robustness of findings. All costs and QALY were discounted at 3% per year.

**Results:**

In the base case analysis, an 8.5 mm Hg reduction in systolic blood pressure with uRDN compared with SoC alone resulted in lower relative risks of cardiovascular events and additional life years (15.00 vs 14.29) and QALY (12.01 vs 11.42). Using a procedure cost of $23,000, total lifetime costs were higher with the uRDN procedure (uRDN plus SoC: $110,476 vs SoC alone: $102,875), resulting in an incremental cost-effectiveness ratio of $12,900 per QALY. Sensitivity and scenario analyses demonstrate that the findings were robust to changes in key model inputs including a systolic blood pressure reduction with uRDN from –5.0 to –9.6 mm Hg.

**Conclusions:**

Evaluated against conventional cost-effectiveness thresholds in the US, the addition of uRDN is estimated to offer a cost-effective approach alongside lifestyle modification and antihypertensive medications for patients with resistant hypertension.

## Introduction

Hypertension is a leading preventable cause of cardiovascular (CV) disease and mortality, affecting nearly 1 in 2 adults in the United States.^1^ Among adults with hypertension, 3 in 4 have uncontrolled hypertension, defined as systolic blood pressure (SBP) ≥140 mm Hg or diastolic blood pressure ≥90 mm Hg while either untreated or on antihypertensive medication.^2^^,^^3^ Moreover, if the patient has been treated with 3 classes of antihypertensive medication, including a diuretic, a renin-angiotensin-system blocker, and a calcium channel blocker at a maximally tolerated dose, their uncontrolled hypertension is classified as resistant hypertension (rHTN).^4^ rHTN is estimated to have a prevalence across the globe of 10% to 15% of the treated hypertensive population.^5^ In 2017, the annual economic burden associated with hypertension in the US, including the cost of health care services, medications, and productivity loss, was estimated between $131 and 198 billion.^6^

Currently, patients with rHTN have limited options to achieve better control of their blood pressure substantially increasing their risk of death and adverse CV outcomes. Ultrasound renal denervation (uRDN) is a catheter-based, minimally invasive procedure that disrupts renal sympathetic nerve activity. In the RADIANCE-HTN TRIO trial, a contemporary multicenter, randomized, sham-controlled trial, patients receiving uRDN reported a mean office SBP reduction at 2 months follow-up of –8.5 mm Hg that persisted up to 36 months postprocedure.^7^^,^^8^ Overall, a long-term reduction in office SBP of 5 mm Hg is associated with clinically meaningful reductions in major CV events, with a linear association between the magnitude of SBP and CV risk reduction.^9^^,^^10^

Previous studies have evaluated the reduction in CV events following the reduction in SBP and demonstrated the cost effectiveness of renal denervation in patients with rHTN.^11^, ^12^, ^13^, ^14^, ^15^ We already reported the cost-utility of uRDN compared with standard of care (SoC) alone from the perspective of the United Kingdom (UK) health care system.^14^ This prior economic evaluation reported a base case incremental cost-effectiveness ratio (ICER) of £5600 per quality-adjusted life year (QALY) below the cost-effectiveness threshold used by the National Institute for Health and Care Excellence of £20,000 to £30,000 per QALY.

Building on the previously published UK-based analysis, this study aimed to provide an assessment of the cost-utility of uRDN from the perspective of the US health care system.

## Methods

### Study design

This economic evaluation was conducted from the perspective of the US health care system. US-based health care costs were sourced from published literature, with clinical inputs from the RADIANCE-HTN TRIO trial.^7^ A state-transition (Markov) model, based on our previously published cost-utility analysis from the perspective of the UK health care system, was employed to determine the effect of treatment with uRDN plus SoC versus SoC alone.^14^ A lifetime horizon was used to measure all potential costs and outcome effects of uRDN, with an annual discount rate of 3.0%.^16^ The analysis was conducted in accord with the Consolidated Health Economic Evaluation Reporting Standards (CHEERS) statement.^17^

### Patient population

The base case analysis used the rHTN population based on the RADIANCE-HTN TRIO trial inclusion and exclusion criteria.^7^ The trial population included 136 patients aged 18 to 75 years from 28 tertiary centers in the US and Europe, whose office SBP was ≥140/90 mm Hg despite treatment with a SoC that included ≥3 antihypertensive medications, including a diuretic, reflecting current guideline recommendations.^18^ After 4 weeks of standardized therapy on a single pill triple combination therapy of a thiazide, an angiotensin receptor blocker, and a calcium channel blocker, participants who remained with rHTN on ambulatory blood pressure monitoring were randomly assigned to receive uRDN (n = 69) or a sham procedure (n = 67).

### Model structure

**Figure 1:**
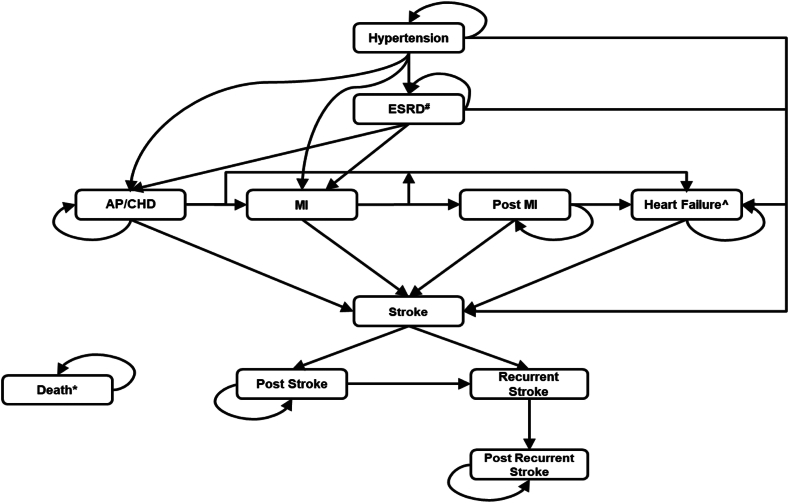
**Markov model structure.** AP, angina pectoris; CHD, coronary heart disease; ESRD, end-stage renal disease; MI, myocardial infarction.

The Markov model (Figure 1) was updated with inputs to reflect the US perspective of this analysis. The model simulated outcomes of 5 CV events resulting from rHTN, including angina pectoris or coronary heart disease, end-stage renal disease (ESRD), myocardial infarction, heart failure, and stroke. All patients started in the hypertension state and moved to a different health state when a CV event occurred. To characterize the elevated long-term risks and reduced utility for recurrent stroke patients, a recurrent stroke health state was modeled separately. In addition, memory states were added to capture higher costs and reduced utility in ESRD patients who subsequently experienced a CV event (eg, ESRD patients who later have a stroke). Death was an absorbing health state, which could be entered from any other state. The model used a monthly cycle, and a half-cycle correction was applied.

Risk equations based on the Framingham and Prospective Cardiovascular Münster studies were used to predict the incidence of CV events.^19^^,^^20^ Relative risks associated with the downstream effects of SBP reduction were based on a meta-analysis of 55 randomized clinical trials involving 195,267 patients with hypertension.^21^

### Clinical and health utility inputs

Key model inputs are summarized in Table 1^7^^,^^16^^,^^22^, ^23^, ^24^, ^25^, ^26^, ^27^, ^28^, ^29^, ^30^, ^31^, ^32^, ^33^, ^34^, ^35^, ^36^ and Supplemental Table S1. Based on aggregate-level data from the RADIANCE-HTN TRIO trial, the mean baseline office SBP was 155.3 mm Hg, and a mean reduction of 8.5 mm Hg was observed in the uRDN arm at 2 months follow-up.^7^ In accordance with the previous economic evaluation of uRDN,^12^ the base case analysis assumed no SBP reduction associated with SoC because a sham intervention would not be performed in real-world settings. Sensitivity analyses were performed varying the treatment effect from reduction in blood pressure from 4.5 mm Hg to 12.5 mm Hg as described below.

**Table 1:** Summary of key model input parameters.^a^^,^^b^

Parameter	Value	Distribution	Standard Error	Source
Age, y	52.6	Normal	0.72	RADIANCE-HTN TRIO^7^
Female sex, %	20	Beta	0.03	RADIANCE-HTN TRIO^7^
Weight, kg	99.9	Normal	1.82	RADIANCE-HTN TRIO^7^
Height, m	1.7	Normal	0.01	RADIANCE-HTN TRIO^7^
Body mass index, kg/m^2^	32.6	Normal	0.49	RADIANCE-HTN TRIO^7^
Baseline and treatment size				
Baseline SBP, mm Hg	155.3	Normal	1.46	RADIANCE-HTN TRIO^7^
SBP treatment effect, mm Hg	–8.5	Normal	2.39	RADIANCE-HTN TRIO^7^
Discount rate				
Discount rate (QALY)	3%	–	–	Neumann et al,^16^ 2016
Discount rate (costs)	3%	–	–	Neumann et al,^16^ 2016
Costs				
uRDN procedure	$23,000	Gamma	$2300	ReCor Medical
Stroke (acute)	$29,376	Gamma	$2938	Johnson et al,^22^ 2016; Salata et al,^23^ 2016
Stroke (monthly maintenance)	$2656	Gamma	$266	Johnson et al,^22^ 2016; Henk et al,^24^ 2015
MI (acute)	$20,225	Gamma	$2023	Krumholz et al,^25^2014
MI (monthly maintenance)	$576	Gamma	$58	Ito et al,^26^2015
AP/CHD (acute)	$10,240	Gamma	$1024	Nicholson et al,^27^ 2016
AP/CHD (monthly maintenance)	$613	Gamma	$61	Fearon et al,^28^ 2018
HF (acute)	$2244	Gamma	$224	Urbich et al,^29^ 2020
HF (monthly maintenance)	$2244	Gamma	$224	Urbich et al,^29^ 2020
ESRD (acute)	$7519	Gamma	$752	United States Renal Data System,^30^ 2021
ESRD (monthly maintenance)	$7519	Gamma	$752	United States Renal Data System,^30^ 2021
Health utilities				
Hypertension	1.00	Beta	0.1	Assumption
AP/CHD (unstable)	0.91	Beta	0.04	Ward et al,^31^ 2007; Glasziou et al,^32^ 2007
AP/CHD (stable)	0.96	Beta	0.08	Goodacre et al,^33^ 2004; Glasziou et al,^32^ 2007
MI (mo 0-6)	0.90	Beta	0.02	Schlaich et al,^34^2013
MI (mo 7+)	1.00	Beta	0.1	Ward et al,^31^2007; Goodacre et al,^33^2004
HF	0.88	Beta	0.08	Comin-Colet et al,^35^ 2013
Stroke	0.85	Beta	0.08	Ward et al,^31^ 2007
ESRD	0.92	Beta	0.23	Gorodetskaya et al,^36^ 2005

AP, angina pectoris; BP, blood pressure; CHD, coronary heart disease; ESRD, end-stage renal disease; HF, heart failure; MI, myocardial infarction; QALY, quality-adjusted life year; uRDN, ultrasound renal denervation.

a All utilities are corrected for age.

b Costs in US dollars are inflated to 2022 price level.

Other clinical inputs were derived from published literature. With the exception of ESRD, all CV events were modeled using risk equations, which estimate the probability of transitioning between different health states over time as a function of several CV risk factors, including office SBP. The model estimated the risk of stroke, heart failure, and angina pectoris or coronary heart disease using risk equations from the Framingham Heart Study.^19^ The risk of myocardial infarction was modeled using the risk equation from the Prospective Cardiovascular Münster study.^20^ Because there were no available risk equations for ESRD that incorporated office SBP, the risk of ESRD was modeled using a general population risk by age with an applied relative risk based on office SBP. Utilities for specific health states and age-related decrements were derived from multiple sources.^11^^,^^37^ General population mortality was based on the 2020 United States life tables in the Centers for Disease Control and Prevention National Vital Statistics Reports.^38^ Further details about risk equations and clinical inputs are provided in Supplemental Tables S2-S4.

### Cost inputs

Major CV event costs were sourced from a recently published study evaluating the cost-effectiveness of radiofrequency RDN for uncontrolled hypertension from a US health care system perspective.^14^ The event costs reported in this study were aligned with our model’s cost structure, which includes an acute cost for the first month postevent and monthly follow-up costs for ongoing care. The adapted event costs are reported in Table 1. Drug acquisition costs were derived from the “Red Book,” a tool developed by Micromedex (a health care brand of IBM Watson Health), which includes pricing information on US Food and Drug Administration (FDA)-approved drug products.^39^ The cost of the uRDN procedure was $23,000 (from ReCor Medical). Monthly monitoring costs are calculated with an assumption of 2 doctor visits per year. Direct device-related adverse events costs were not considered, as the uRDN procedure has a low complication rate (eg, major access site complications requiring intervention in only 1% of RDN patients^7^). All cost inputs used in this model are inflated to 2022 US dollars using the Consumer Price Index for Medical Care.^40^

### Data analysis

Results are reported as ICER, the ratio of the mean difference in costs, and the mean difference in QALY between uRDN plus SoC vs SoC alone.

To supplement the base case analysis, 1-way, and probabilistic sensitivity analyses were performed to account for any uncertainty in the model inputs. The 1-way sensitivity analysis involved sequential calculations of plausible minimum and maximum values for each model parameter. In probabilistic sensitivity analysis, each parameter was simultaneously varied according to predefined distributions, with results presented for 1000 iterations. Most variables were assumed to have a normal distribution (see Table 1), except for proportions, probabilities, and utility estimates, which were all varied using a beta distribution. Hazard ratios, as well as the costs of the uRDN procedure, antihypertensive medications, and CV events, were varied using a gamma distribution.

Several scenario analyses were used to explore the impact of alternative model inputs and assumptions on the results. These included the following: (1) the sham-subtracted clinical effect size from the RADIANCE-HTN TRIO trial (–5.0 mm Hg),^7^ (2) the clinical effect size among the US participants of the RADIANCE-HTN TRIO trial (–9.6 mm Hg),^7^ (3) alternative baseline SBP value from SPYRAL HTN-ON MED trial that aligns with a recent US cost-effectiveness publication of catheter-based radiofrequency (163 mm Hg),^15^ (4 and 5) applying SBP and CV event risk risks from Ettehad et al^10^ or Rahimi et al^41^ (Blood Pressure Lowering Treatment Trialists' Collaboration), and (6) a reduced model time horizon of 20-years.^42^

## Results

### Base case results

The base case analysis demonstrated that patients treated with uRDN experience lower relative risks of adverse CV events and improvements in life years and QALY gained compared with patients treated with the SoC alone (see Supplemental Tables S2-S4). Over the lifetime horizon, uRDN patients gain a mean of 15.00 life years and 12.01 QALY, compared to SoC patients who gain a mean of 14.29 life years and 11.42 QALY. The uRDN procedure is associated with higher lifetime costs compared to the SoC alone ($110,476 vs $102,875 per patient). This results in an ICER of $12,900 per QALY (Table 2).

**Table 2:** Base case cost-effectiveness results for uRDN plus SoC vs SoC alone.

Treatment	LY	QALY	Costs	ΔLY	ΔQALY	ΔCosts	ICER ($/LY)	ICER ($/QALY)
uRDN plus SoC	15.00	12.01	$110,476	0.71	0.59	$7600	$10,645	$12,900
SoC alone	14.29	11.42	$102,875	–	–	–	–	–

QALY and LY are discounted at 3% annually.

$, US dollar; ICER, incremental cost-effectiveness ratio; LY, life year; QALY, quality-adjusted life year; SoC, standard of care; uRDN, ultrasound renal denervation; Δ, difference between uRDN plus SoC vs SoC alone.

### One-way sensitivity analysis

**Figure 2:**
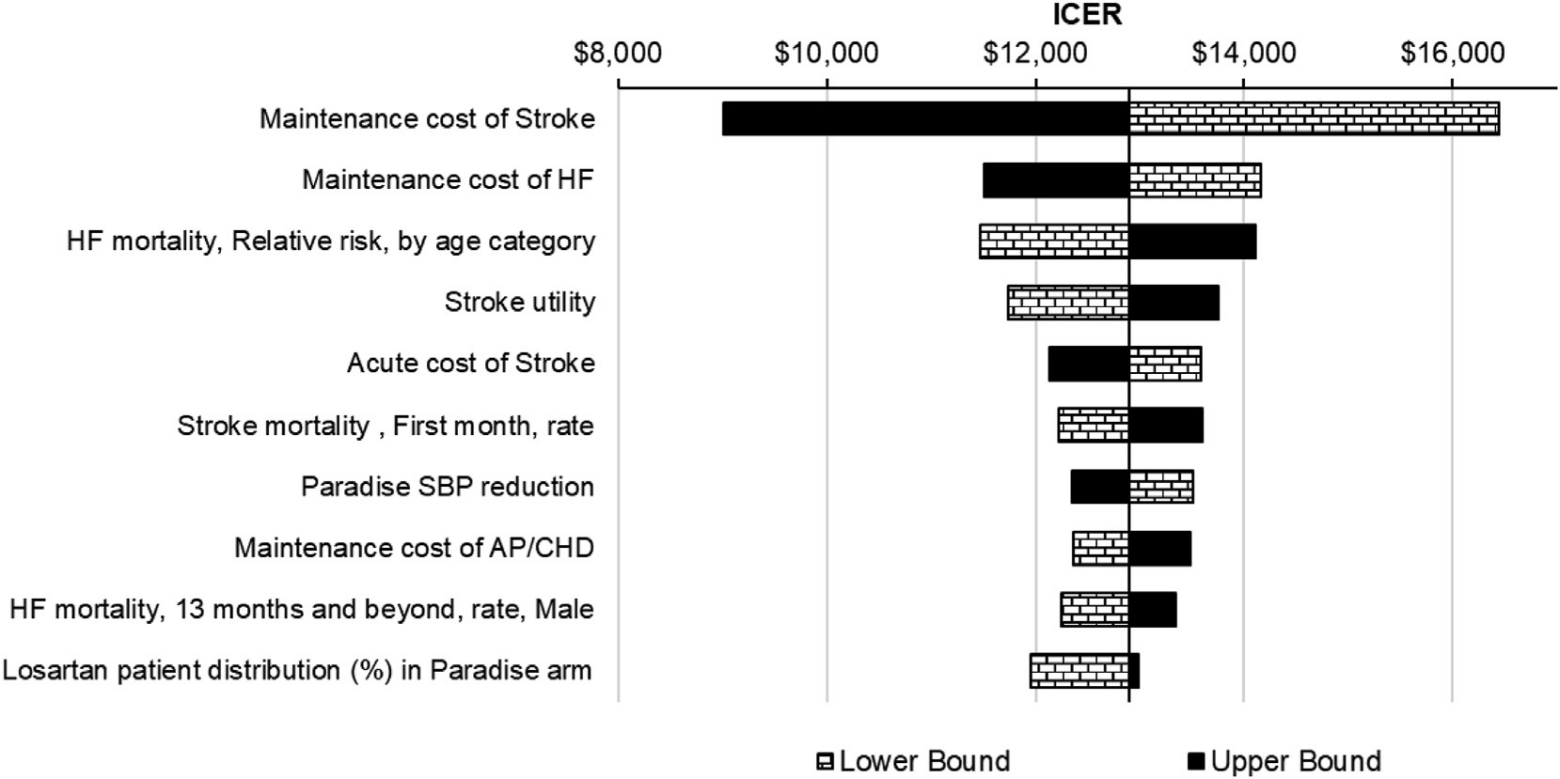
**Tornado diagram (1-way sensitivity analysis) of the incremental cost-effectiveness ratio (ICER) for ultrasound renal denervation plus standard of care vs standard of care alone.** AP, angina pectoris; CHD, coronary heart disease; HF, heart failure; SBP, systolic blood pressure.

These base case findings were robust to changes in key model inputs, including costs, relative risks, health state utilities, and mortality associated with key CV events. The tornado diagram in Figure 2 details the sensitive parameters and the ICER for the upper and lower bound values of these parameters. The most impactful 3 parameters were the maintenance costs of stroke and the relative risk of heart failure mortality. Despite uncertainty around these parameters, uRDN remained highly cost effective (ie, under the cost-effectiveness threshold of $50,000 per QALY) when inputs were individually varied.

### Scenario assessments

Several scenario assessments were also performed to assess the robustness of model results to changes in key assumptions and inputs (Table 3).^7^^,^^10^^,^^15^^,^^41^ Applying the RADIANCE-HTN TRIO sham-subtracted SBP effect size (–5.0 mm Hg) (scenario 1), using the alternative SBP or CV risk equations of Ettehad et al^10^ or Rahimi et al^41^ (scenarios 4 and 5), and a shorter model time horizon (scenario 6) all led to higher ICER than seen for base case analysis. Using the RADIANCE-HTN TRIO trial uRDN change in treatment effect size seen in US participants alone (–9.6 mm Hg) (scenario 2) and using baseline office SBP from SPYRAL HTN-ON MED (163 mm Hg) (scenario 3), resulted in a similar ICER to the base case. All scenario ICER remained below the cost-effectiveness threshold of $50,000.

**Table 3:** Scenario analyses for uRDN plus SoC vs SoC alone.

Scenario		ΔLY	ΔQALY	ΔCosts	ICER ($/LY)	ICER ($/QALY)
1	Sham-subtracted effect size (–5.0 mm Hg)	0.412	0.340	$13,981	$33,919	$41,125
2	Effect size in US patients in RADIANCE-HTN TRIO (–9.6 mm Hg)^7^	0.811	0.669	$5582	$6883	$8340
3	Baseline office SBP based on SPYRAL HTN-ON MED (163 mm Hg)^15^	0.772	0.636	$7803	$10,105	$12,278
4	Applying RR from Ettehad et ​al^10^	0.450	0.373	$12,664	$28,114	$33,953
5	Applying HR from Rahimi et ​al^41^ (BPLTTC)	0.367	0.308	$14,165	$38,638	$46,049
6	Time horizon of 20 y	0.338	0.311	$9029	$26,681	$29,015

$, US dollar; HR, hazard ratio; ICER, incremental cost-effectiveness ratio; LY, life year; QALY, quality-adjusted life year; RR, relative risk; SBP, systolic blood pressure; SoC, standard of care; uRDN, ultrasound renal denervation; Δ, difference between uRDN plus SoC vs SoC alone.

### Probabilistic sensitivity analysis

**Figure 3:**
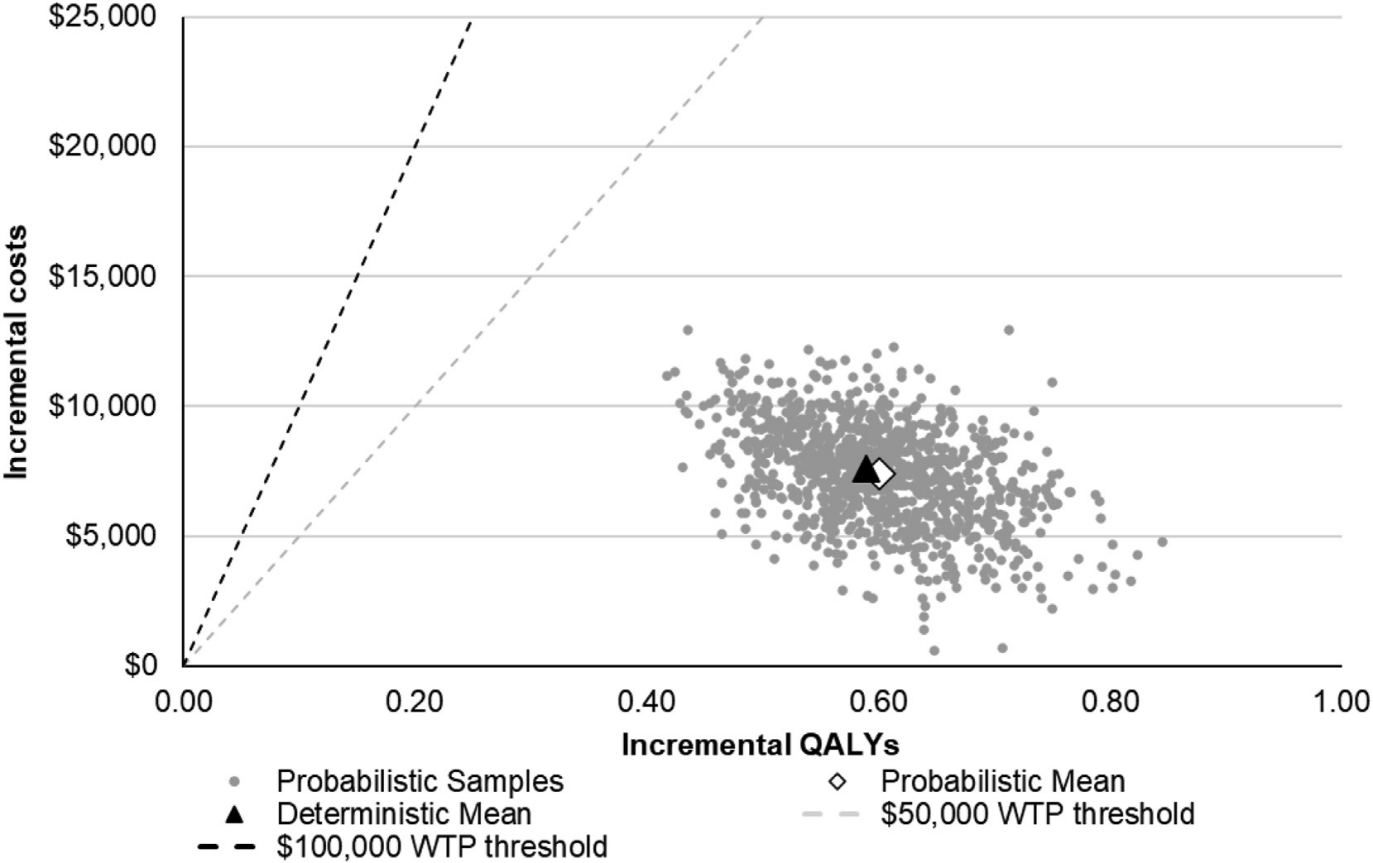
**Probabilistic sensitivity analysis of ultrasound renal denervation plus standard of care vs standard of care alone.** QALY, quality-adjusted life year; WTP, willingness-to-pay.

**Figure 4:**
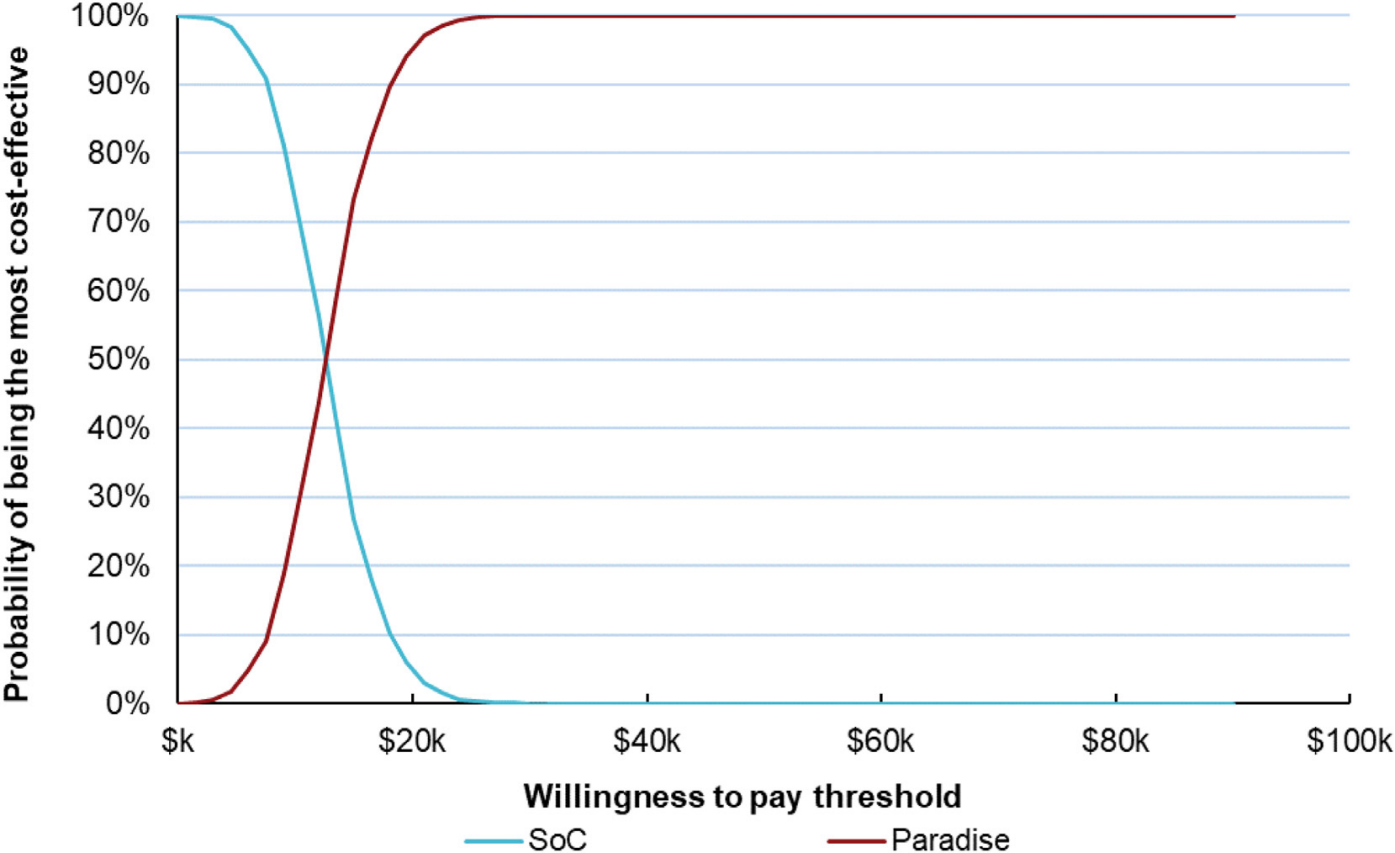
**Cost-effectiveness acceptability curve for ultrasound renal denervation plus standard of care (SoC) vs standard of care alone.**

Figure 3, Figure 4 show the probability that uRDN is cost effective in the probabilistic sensitivity analysis as a function of the cost-effectiveness threshold. At a threshold of $15,000 per QALY gained, 73.2% of iterations found the procedure cost effective. As shown in Figure 4, 100% of iterations in the probabilistic sensitivity analysis found uRDN cost-effective relative to a threshold of $50,000 per QALY.

## Discussion

Evidence from contemporary clinical studies using rigorous trial designs, demonstrating both safety and efficacy^7^^,^^8^ has led to approval of uRDN by the US FDA as an adjunctive treatment to traditional antihypertensive therapy for patients with uncontrolled hypertension despite ongoing medications.^43^ The place of uRDN in the treatment pathway is also a recommendation of recent international clinical society guidelines.^44^^,^^45^ This economic evaluation demonstrates the provision of uRDN in the US health care system to be a cost-effective treatment strategy. Our base case results over the patient’s lifetime show that the addition of uRDN provides a mean gain of 15.00 life years and 12.01 QALY in comparison to 14.29 life years and 11.42 QALY with SoC alone. Based on a procedure cost of $23,000, this health gain of uRDN corresponds to an ICER of $12,900 per QALY, well below the $50,000 to $100,000 per QALY threshold typically considered as the threshold for value-based health technologies.^46^^,^^47^ This conclusion was robust to sensitivity and scenario analyses, all of which produced ICER that all remained below the threshold of $50,000 per QALY.

The only previously published cost-effectiveness analysis of uRDN, was also conducted by our group^14^ and was based on the Markov model used in the present study. Consistent with the present study, it was concluded that uRDN was a highly cost-effective intervention with a >99% probability that it falls below the UK cost-effectiveness threshold of £20,000 to £30,000 (∼$25,000 to ∼$40,000) per QALY. The recently published economic evaluation undertaken by Kandzari et al^15^ shows catheter-based radiofrequency RDN to be also cost effective. Undertaken from the perspective of the US health care system, this analysis undertook 2 base case analyses using SBP data from either the SPYRAL HTN-ON MED (RDN in the presence of antihypertensive medications) or SPYRAL HTN-OFF MED (RDN in the absence of antihypertensive medication) trials. ICER of $32,732 and $25,521 per QALY respectively were reported.

In accordance with our previous modeling paper, which examined the cost effectiveness of uRDN from the perspective of the UK health care system, this study has several strengths.^14^ First, it uses contemporary high-quality trial evidence (RADIANCE-HTN TRIO trial^7^) and is based on a validated, peer-reviewed Markov decision analytic model.^14^ Second, this study undertakes extensive sensitivity and scenario analyses to elaborate on the findings of the base case analysis and increases the certainty of the central findings across a range of different scenarios. Third, it uses clinical and cost parameters that directly reflect the US health care system and have previously been published in association with an evaluation of another renal denervation modality.^15^

However, we recognize there are also potential limitations in our analysis albeit many of these are in common with prior model-based economic evaluations of antihypertensive medications and renal denervation therapies.^48^ First, a central assumption is that the observed reduction in SBP with uRDN in the clinical trial setting will translate into future reductions in CV morbidity and mortality. However, the authors note that trial-based SBP change is a well-accepted surrogate end point for CV events by regulators around the globe and therefore it is felt that it can be defended. SBP is listed in the FDA’s current table of surrogate end points that have been approved as the basis of supporting approval and patient access to new medical treatments.^49^ Meta-analyses of randomized trials of antihypertensive medications have shown SBP to be a valid surrogate end point for clinical events, including reduction of stroke risk.^50^ Furthermore, the predictive effect of SBP is largely consistent across classes of antihypertensive drugs (with the exception of beta-blockers) providing a basis for the hypothesis that the protective effect of SBP reduction can also be applied to RDN. Nevertheless, future analyses of the validity of SBP as a surrogate end point should consider contemporary RDN evidence through access to individual patient data from recent and future trials. Second, this decision-analysis–based modeling assumes that the antihypertensive benefits of RDN continue over the patient’s lifetime. The durability of SBP impact of modulating renal sympathetic nerve activity through RDN modalities is supported by published long-term follow-up to 10 years.^51^ Clinical data from the RADIANCE-HTN TRIO trial show the persistence of the SBP-lowering effect of uRDN out to 36 months.^8^ Scenario analyses presented in this study offer increased certainty of the findings and the central conclusion of cost-effectiveness. Although the base case analysis applied CV predictions based on Thomopoulos et al,^21^ we also demonstrated that our results were robust to the application of the CV risk equations of Ettehad et al^10^ and Rahimi et al.^41^ In addition to the base case analysis over the patient’s lifetime, we also demonstrate that the use of uRDN over the shorter time horizon of 20 years remains a cost-effective treatment strategy. Although it can be argued that sham is the most appropriate comparator to obtain an unbiased estimate of RDN efficacy, sham control does not reflect true clinical practice where the real-world clinical effectiveness of the addition of RDN would be assessed in comparison to SoC of antihypertensive medications only. Our use of the reduction in SBP from baseline to 2 months with RDN from the RADIANCE-HTN TRIO trial aims to provide a real-world proxy for the anticipated impact of the introduction of uRDN into the clinical pathway for rHTN in the United States. Irrespective of this, scenario analysis using the lower sham-subtracted SBP reduction of –5.0 mm Hg demonstrated uRDN to remain cost effective. Third, model parameters were sourced using a targeted rather than systematic literature review. To preserve consistency with the previously published UK-based model, we retained well-established clinical parameters unlikely to vary across settings while conducting a targeted search for US-specific inputs. Event cost inputs were aligned with a recently published US-based RF RDN cost-effectiveness study, which synthesized several relevant underlying cost inputs.^15^ Although a systematic literature review was not conducted, the model’s sensitivity to key input values, including clinical and cost parameters, was thoroughly vetted.

## Conclusion

In support of contemporary uRDN clinical trial evidence demonstrating clinical efficacy and safety, this analysis shows that adding uRDN to the existing landscape of lifestyle modification and antihypertensive medications for people with rHTN is a cost-effective strategy in the US health care setting (Central Illustration). As a one-time, minimally invasive intervention, uRDN addresses a critical unmet need for patients, clinicians, and policymakers given the recognized clinical consequences of uncontrolled hypertension.

**Central Illustration:**
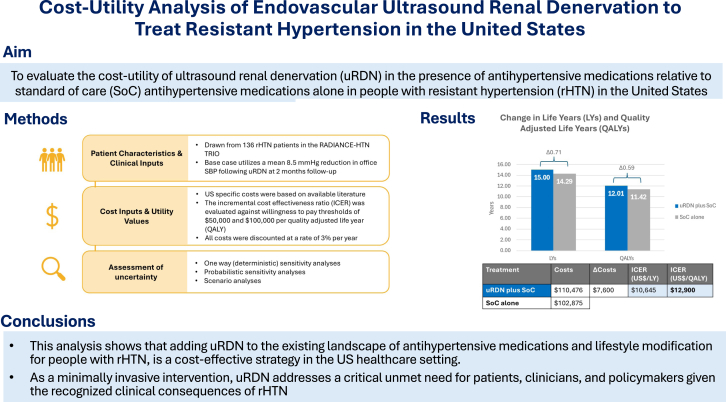
**Cost-utility analysis of endovascular ultrasound renal denervation to treat resistant hypertension in the UnitedStates.**

## Footnotes

To access the supplementary material accompanying this article, visit the online version of the *Journal of the Society for Cardiovascular Angiography & Interventions* at 10.1016/j.jscai.2025.103601.
