# Supplementary material for: Cost-Utility Analysis of Endovascular Ultrasound Renal Denervation to Treat Resistant Hypertension in the United States
Source: J Soc Cardiovasc Angiogr Interv. 2025 Jun 17;4(6):103601. doi: 10.1016/j.jscai.2025.103601 (PMC12230492; doi:10.1016/j.jscai.2025.103601)
Supplement: Supplemental Tables [file mmc1.docx]

**SUPPLEMENT**

Supplemental Table S1. Summary of event risk and mortality inputs

| **Health State** | **Event risks** |
| --- | --- |
| AP/CHD risk | Weibull distribution of age, sex, SBP, post-menopausal status, total cholesterol, HDL, triglycerides, interaction term for antihypertensive therapy and SBP, diabetes mellitus, smoking, and alcohol use |
| ESRD risk | We fitted a model based on SBP dependent hazard ratios |
| HF risk | Exponential distribution of age, sex, SBP, left ventricular hypertrophy, vital lung capacity, coronary heart disease, valve disease, diabetes mellitus, cardiomegaly, heart rate.  Risk of developing heart failure in patients with an MI: 23.1%  Hazard ratio for developing heart failure in patients with (a history of) AP: 1.35 |
| MI risk | Exponential distribution of age, SBP, triglycerides, HDL, LDL, gamma glutamyl transferase, smoker, diabetes, family history. Relative risk of MI in AP/CHD patients based on age group; 15-44, 0.261; 45-54, 0.630; 55-64, 1; 65-74, 1.371; 75+, 1.826. |
| Stroke risk | Initial stroke: Exponential distribution of age, sex, SBP, medication use, CVD, LVH, smoker, atrial fibrillation, diabetes mellitus. Relative risk of stroke in ESRD: Ethnicity and gender-adjusted relative risk: female from non-African descent, 9.7; female from African descent, 6.2; male from non-African descent, 6.1, male from African descent, 4.4.  Recurrent stroke: 0.679% in the first cycle after stroke, slowly decreasing over time. |
| Relative risks | RR per 10 mmHg: AP/CHD: 0.78, Stroke: 0.63, HF: 0.54 |
| **Health State** | **Mortality** |
| Hypertension | 2020 US general population mortality |
| AP/CHD | Annual rate per age group: 35-44: 0.46% (male); 0.25% (female); 45-54: 1.07% (male); 0.62% (female); 55-64: 1.84% (male); 1.20% (female); 65-74: 3.27% (male); 2.51% (female); 75-84: 10.59% (male); 9.64% (female) |
| ESRD | Annual rate per age group:  20-44: 5.75%; 45-64: 11.12%; 65-74: 20.42%; 75+: 35.54% |
| MI | For the first month, a rate per age group is applied; 35-44: 1.50%; 45- 54: 3.40%; 55-64: 7.30%; 65-74: 15.90%; >=75: 29.50%  Beyond the first month, a probability per cycle is applied based on SBP levels:  <120 mmHg, 0.168%; 120-139 mmHg, 0.195%; 140-159 mmHg, 0.256%; ≥160 mmHg, 0.307%.  A HR is applied to correct for age over 60: 60-69 years, 1.28; ≥70 years, 2.46. |
| HF | Rate based on time since onset and gender: 30 days: 6.00% (male); 4.00% (female); Year 1: 21.00% (male); 17.00% (female); Year 2 and following: 50.00% (male); 46.00% (female)  Age dependent HRs are applied to correct for age: <50 years, 1; 50-54 years, 1.03; 55-59 years, 1.02; 60-64 years, 1.28; 65-69 years, 1.72; 70-74, 2.20; 75-79, 2.86; ≥80 years, 3.68. |
| Stroke | First month: Rate of 12.60% Long-term stroke: HR vs background mortality: 2.30 Acute risk post-MI: 2.27 Long-term risk post-MI: 2.99 Relative risk post-HF: 2.189 |

**Abbreviations:** AP: angina pectoris; BMI: body mass index; CHD: coronary heart disease; CVD: cardiovascular disease; ESRD: end-stage renal disease; HDL: high-density lipoprotein cholesterol; HF: heart failure; HR: hazard ratio; HTN: hypertension; LDL: low-density lipoprotein; LVH: left ventricular hypertrophy; MI: myocardial infarction; SBP: systolic blood pressure; SoC: standard of care; RR: relative risk.

Supplemental Table S2. Lifetime cardiovascular events per arm

1. Cardiovascular events occurring over lifetime horizon.

| **Event** | **uRDN plus SoC** | **SoC** | **Incremental impact** |
| --- | --- | --- | --- |
| ESRD | 0.63% | 0.66% | 0.03% |
| AP | 26.13% | 24.61% | -1.51% |
| MI | 22.84% | 21.55% | -1.29% |
| HF | 17.04% | 21.19% | 4.15% |
| Stroke | 37.27% | 44.92% | 7.65% |
| Recurrent stroke | 9.42% | 11.58% | 2.16% |
| Mortality | 99.58% | 99.67% | 0.09% |

1. Life years and quality-adjusted life years per health state.

| **Event** | **uRDN plus SoC** | | **SoC** | |
| --- | --- | --- | --- | --- |
|  | **LYs** | **QALYs** | **LYs** | **QALYs** |
| Hypertension | 11.32 | 9.37 | 10.38 | 8.63 |
| ESRD | 0.02 | 0.01 | 0.02 | 0.01 |
| AP | 1.36 | 1.01 | 1.14 | 0.85 |
| MI | 0.55 | 0.43 | 0.50 | 0.39 |
| HF | 0.45 | 0.31 | 0.60 | 0.42 |
| Stroke | 1.04 | 0.68 | 1.32 | 0.88 |
| Recurrent stroke | 0.26 | 0.19 | 0.33 | 0.24 |
| **Total** | **15.00** | **12.01** | **14.29** | **11.42** |

1. Costs accrued per health state.

| **Event** | **uRDN plus SoC** | **SoC alone** |
| --- | --- | --- |
| Hypertension | US$27,429 | US$ 4,269 |
| ESRD | US$1,683 | US$1,610 |
| AP | US$12,625 | US$10,774 |
| MI | US$6,764 | US$6,382 |
| HF | US$12,307 | US$16,440 |
| Stroke | US$40,149 | US$51,231 |
| Recurrent stroke | US$9,518 | US$12,168 |
| **Total** | **US$110,476** | **US$102,875** |

**Abbreviations:** ESRD: end-stage renal disease; AP: angina pectoris; MI: myocardial infarction; HF: heart failure; LY: life year; QALY: quality-adjusted life year; SoC: standard of care; uRDN: ultrasound renal nerve denervation.
